# Participation of Wild Species Genus *Avena* L. (Poaceae) of Different Ploidy in the Origin of Cultivated Species According to Data on Intragenomic Polymorphism of the ITS1-5.8S rRNA Region

**DOI:** 10.3390/plants14101550

**Published:** 2025-05-21

**Authors:** Alexander A. Gnutikov, Nikolai N. Nosov, Igor G. Loskutov, Alexander V. Rodionov, Victoria S. Shneyer

**Affiliations:** 1 N.I. Vavilov Institute of Plant Genetic Resources (VIR), St-Petersburg 190000, Russia; a.gnutikov@vir.nw.ru (A.A.G.); i.loskutov@vir.nw.ru (I.G.L.); 2 Komarov Botanical Institute (BIN RAS), St-Petersburg 197376, Russia; avrodionov@binran.ru (A.V.R.); shneyer@binran.ru (V.S.S.)

**Keywords:** cereals, domestication, genome combinations, grasses, hybridization, molecular phylogeny, next-generation sequencing, polyploidy, rDNA

## Abstract

The possible origin of four cultivated species of the genus *Avena* of different ploidy and different subgenome composition (*A. strigosa*, *A. abyssinica*, *A. byzantina*, and *A. sativa*) from possible wild species was investigated. The region of the internal transcribed spacer ITS1 and the 5.8S rRNA gene in the cultivated species was studied with next-generation sequencing (NGS), and the patterns of occurrence and distribution of the ribotypes were compared among them and with those of the wild species. According to these data diploid, *A. strigosa* is more closely related to the diploid *A. hirtula* than to polyploid oats, and it could have evolved independently of polyploid cultivated species. The tetraploid *Avena abyssinica* could be a cultivated derivative of *A. vaviloviana*. Two hexaploid cultivated species, *A. byzantina* and *A. sativa*, could have a different origin; *A. sativa* could be the cultivated form of *A. fatua*, whereas *A. byzantina* could originate independently. It was found that the oat species with the A and C subgenomes, even with strong morphological and karyological differences, could intercross and pass the further stages of introgression producing a new stable combination of genomes. Our data show that almost all species of *Avena* could form an introgressive interspecies complex.

## 1. Introduction

The genus *Avena* L. (Poaceae) comprises about 30 species. It is one of the most economically important cereals. Oats form a polyploid row from the diploid (2n = 14) to the hexaploid (2n = 42) species, and there are cultivated members in every ploidy group. The cultivated diploids of *Avena* are represented by *A. strigosa* Schreb. and *A. abyssinica* Hochst. ex A.Rich., which are tetraploids, and by *A. byzantina* K.Koch and *A. sativa* L., which are hexaploids. The species of the genus *Avena* are characterized by significant morphological, ecological, and botanical varieties [1], and there is no consensus on the origin of the oat species or their systematic position. Moreover, there is some confusion regarding the naked forms of the diploid and hexaploid cultivated oats. The naked forms of cultivated oats are considered either to be independent species or botanical varieties [2,3].

Karyological studies have revealed four genome types represented in the oat species: A, B, C, and D [4,5,6]. It was found that the initial genome types in *Avena* L. were A and C [4]; whereas the B and D-genomes are derivatives of the A-genomes [7,8,9,10,11,12]. The tetraploids in the genus *Avena* are divided into two groups: AB- and AC-genomes [2,3,7,13,14]. *Avena strigosa* is the species with the As-genome that was considered to be the progenitor of the other A-genome oats [15]. The cultivated tetraploid *A. abyssinica* belongs to the AB-genome group, and there are no cultivated species in the AC-group. *Avena byzantina* and *A. sativa* are both hexaploids (ACD-genome), and apparently originated independently. There is also a contradiction concerning the genome composition of polyploid oats [3,6,16,17,18,19]. For example, previous studies based on cytogenetic analysis have established the AC-genome constitution in such tetraploids as *A. magna* Murphy et Terrell and *A. murphyi* Ladiz., whereas later studies have suggested that these tetraploids have a CD-genome set [18,19]. *Avena magna*, *A. murphyi*, and *A. insularis* Ladiz. are most often treated as the ancestors of the hexaploids [3,6,19,20,21]. In addition, the tetraploid *A. agadiriana* B.R.Baum & Fedak, with the previously defined AB-genome set, is now thought to be the bearer of the DD-genome [6]. Our data, based on the analysis of ITS sequences obtained via NGS, on the contrary, show the presence of A-genome-related sequences in the supposed CD-tetraploids reconstructing their hybridization history as the AC and significant differences in the probable D-genome-sequences compared with the A-genome related rDNA in the CD-species [22]. Most probably, the NGS data simply detects a reorganization process in the genome of the polyploid oats.

The recent studies made on the grass taxa (members of Triticeae and Poeae tribes) revealed some cases of long-distance hybridization, e.g., between geographically separated species and even between the members of different subtribes [23,24,25,26]. The domestication processes in grasses could involve multiple intercrossings as well as differential selection of economically valuable traits, and both can obscure the origin of species. Intraspecific rDNA polymorphism patterns are very useful for tracing hybridization events especially in the cases of introgression [27,28,29,30]. The aim of our study is to try to detect the wild parents of the domesticated oat species, and to check the previous hypotheses of the origin of the cultivated species based on the morphological data.

## 2. Results

The ribotype networks constructed by us show the relationships between the cultivated and the wild oat species. A list of the studied oat species is presented in Table 1. All sequences were sorted into ribotypes with their own set of reads. Major ribotypes are the ribotypes with more than 1000 reads per rDNA pool. We named them according to our previous work concerning wild polyploid species [22] with some changes. A summary of the major ribotypes and their share in the total rDNA pool is presented in Table 2. Minor ribotypes of the studied species are mostly derivatives of the major ribotypes. Primary structure of the major ribotypes is presented in the Table 3.

**Figure 1:**
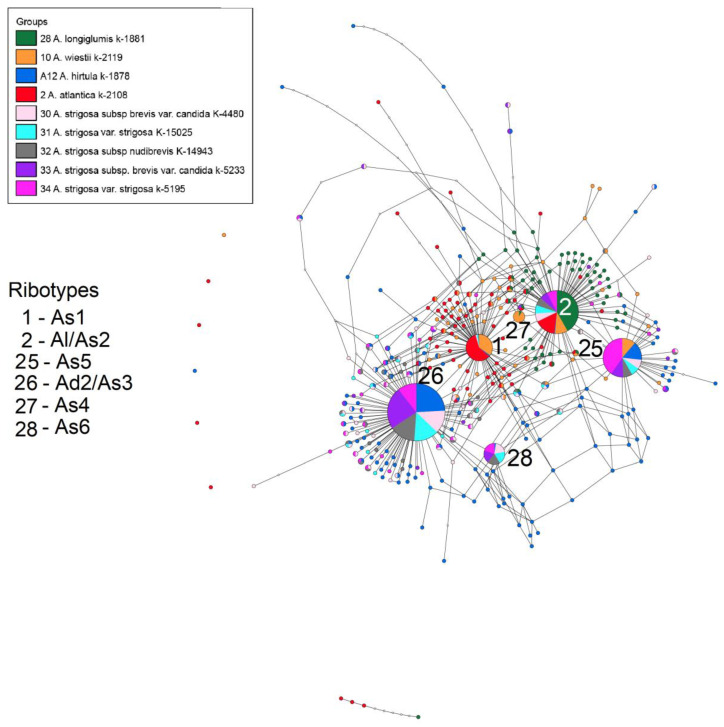
Ribotype network of the cultivated *A. strigosa* and its putative parental taxa. The radius of the circles on the ribotype network is proportional to the percent number of reads for each ribotype, as shown in Table 2. The circles corresponding to the major ribotypes are larger than the others (more than 1000 reads per rDNA pool) and marked with numbers. The smallest circles correspond to ITS1 variants that have been read fewer than 1000 times.

The first network (Figure 1) depicts the possible ribotype phylogeny of the diploid *A. strigosa* (A-genome) and its affinity to the other diploid A-genome bearers. We analyzed five accessions of different varieties of *A. strigosa* along with its wild relatives. The As1-ribotype that is the main ribotype for *A. atlantica* (see [22]) is not common with the major ribotypes of *A. strigosa*, it is shared only with minor fractions of its ribotypes. The main ribotype of *A. strigosa* (all the accessions) is identical to the main ribotype of *A. hirtula* Lag., the second major ribotype of *A. damascena* [22], and the third major ribotype of the As-genome *A. wiestii* (Ad2/As3-ribotype). The second major ribotype of *A. strigosa* subsp. *brevis* (Roth) Husn. var. *candida* Mordv. K-4480 (2412 reads, 11%), *A. strigosa* var. *strigosa* K-15025 (1691 read, 11%), *A. strigosa* subsp. *nudibrevis* (Vavilov) Kobyl. (2282 reads, 11%), and the third major ribotype of *A. strigosa* subsp. *brevis* var. *candida* K-5233 (2962 reads, 12%) is identical with the main ribotype of *A. longiglumis* Durieu (9412 reads, 74%), the second major ribotype of *A. wiestii* Steud. (2901 read, 17%), and the second major ribotype of *A. atlantica* B.R. Baum & Fedak (4571 read, 28%). We named it Al/As2 (because it is the main ribotype in *A. longiglumis*—[22] and in *A. atlantica* that has the As-genome). This ribotype is also the third major ribotype of *A. strigosa* var. *strigosa* (3642 reads, 23%). The As5 ribotype is identical to the second major ribotypes of *A. strigosa* var. *strigosa* (4517 reads, 29%), *A. strigosa* subsp. *brevis* var. *candida* (3707 reads, 15%), and *A. hirtula* (3587 reads, 11%), and the third major ribotypes in *A. strigosa* subsp. *brevis* var. *candida* K-4480 (2325 reads, 11%), *A. strigosa* var. *strigosa* K-15025 (1543 reads, 10%), *A. strigosa* subsp. *nudibrevis* (2135 reads, 11%), and *A. wiestii* (2598 reads, 16%). The As6 ribotype is the fourth major ribotype in *A. strigosa* subsp. *brevis* var. *candida* K-4480 (1703 reads, 8%), *A. strigosa* var. *strigosa* K-15025 (1220 reads, 8%), *A. strigosa* subsp. *nudibrevis* (1628 reads, 8%), *A. strigosa* subsp. *brevis* var. *candida* K-5233 (1995 reads, 8%), and *A. strigosa* var. *strigosa* (2493 reads, 16%).

**Figure 2:**
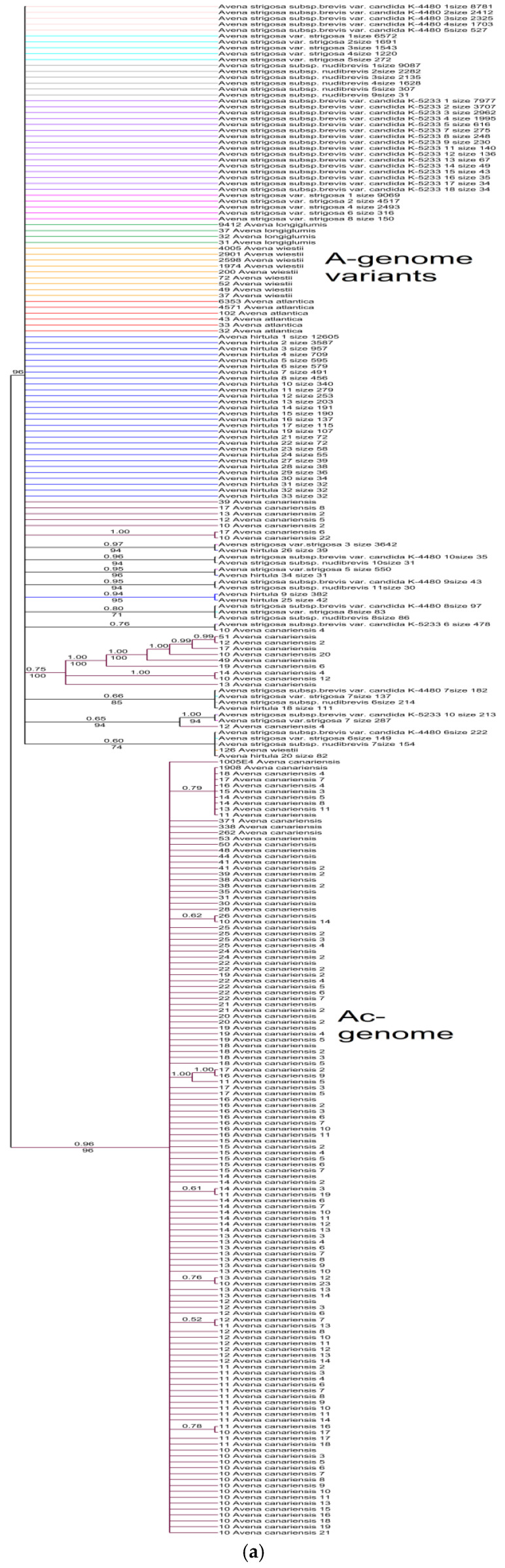

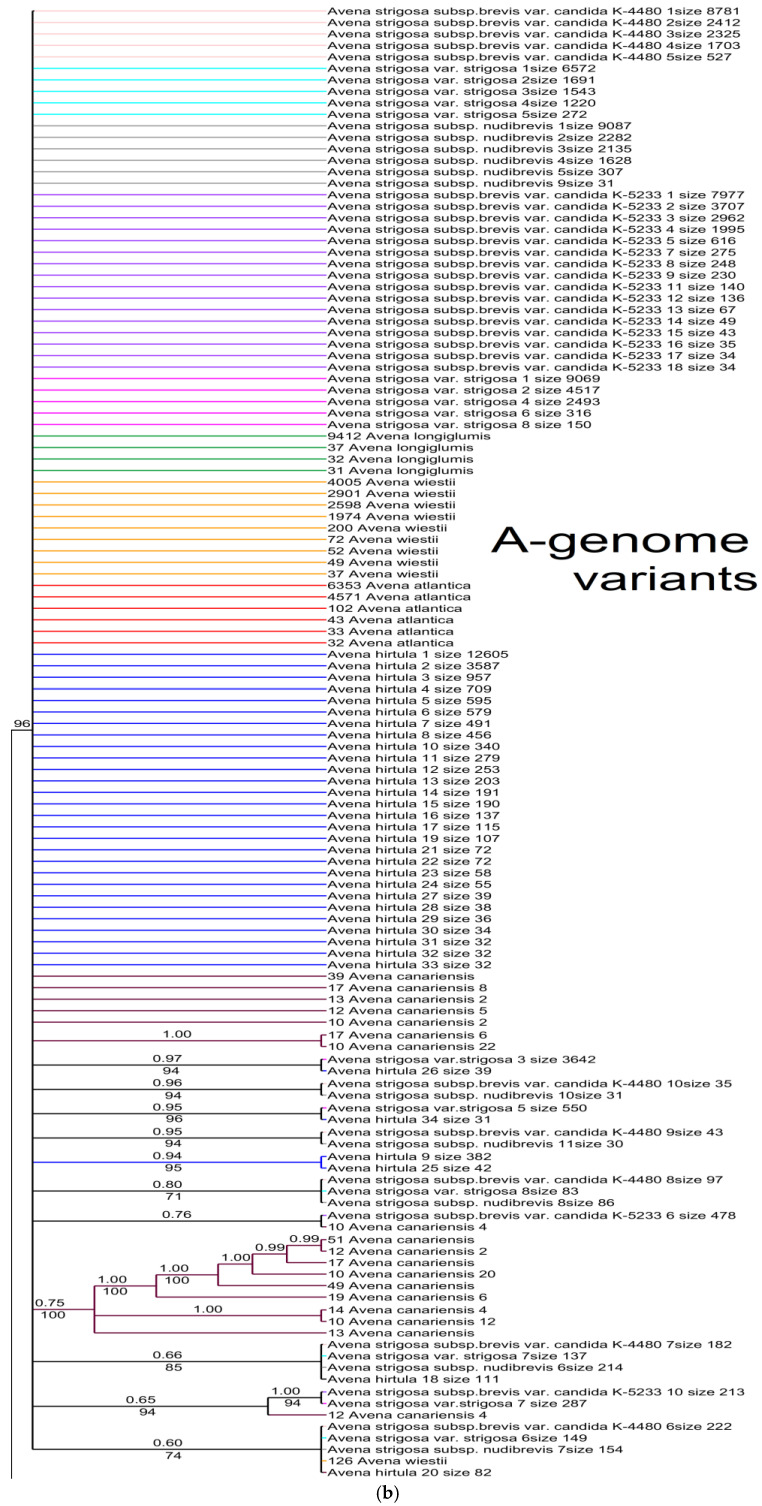

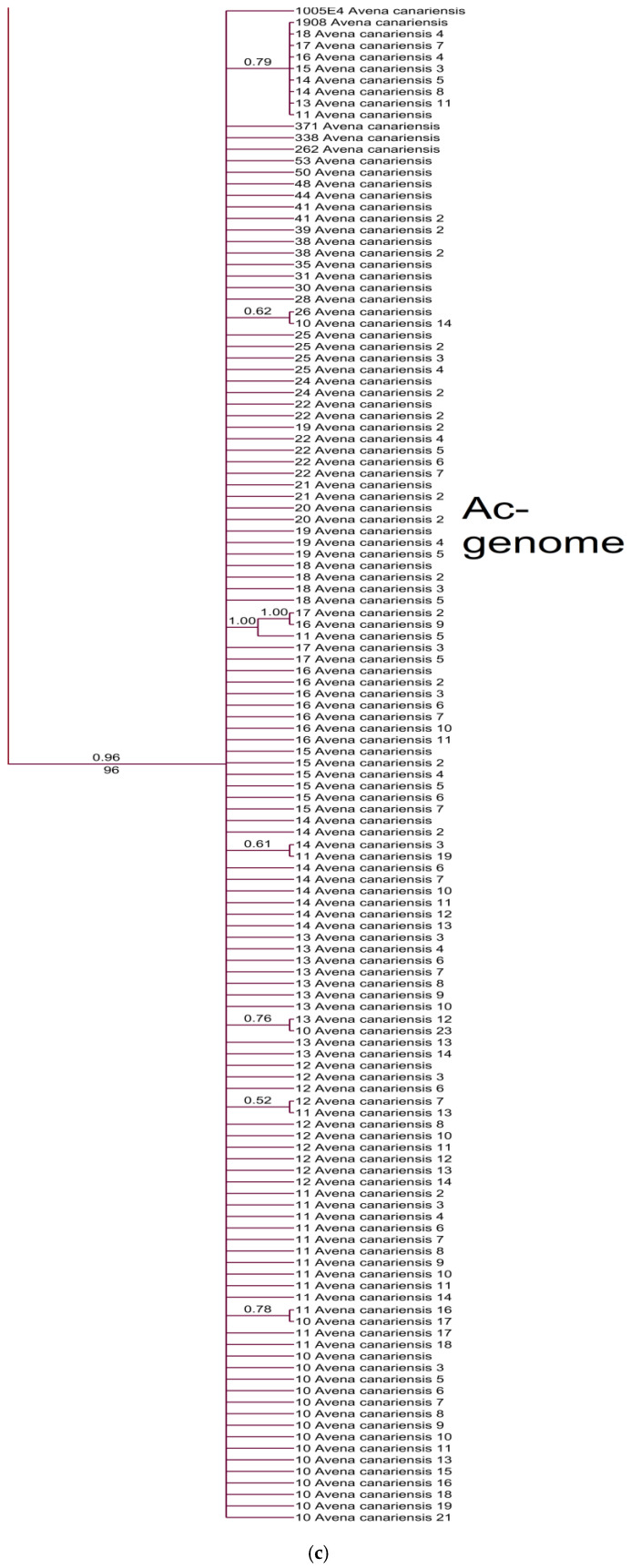
(**a**) Phylogenetic tree of the ribotypes of the cultivated *A. strigosa* and its putative parental species built by the Bayesian inference and the maximum likelihood method. The first index above the branch is the posterior probability in the Bayesian inference, and the second index below the branch is the bootstrap index obtained by the maximum likelihood algorithm. When only one index is shown on the branch, it is the posterior probability. The numbers before the species name indicate the number of reads per rDNA pool. (**b**) A more detailed tree of the A-genome ribotypes (excluding Ac-genome). (**c**) The Ac-genome ribotypes of *A. canariensis* as the separate branch of the A-genome-related ribotypes.

The ribotype tree of *A. strigosa* and its A-genome relatives obtained by the Bayesian inference and the maximum likelihood method shows rather low resolution between the different species (Figure 2). The major ribotypes of *A. canariensis* B.R.Baum, Rajhathy, & D.R.Sampson (Ac-genome) that we chose as the outgroup form a separate clade (PP = 0.96, BS = 96). The minor ribotype fraction of *A. canariensis* forms a separate subclade (PP = 0.75, BS = 100). Only one major ribotype of *A. strigosa* var. *strigosa* (As-genome) falls into the separate subclade with the minor ribotype of *A. hirtula* (PP = 0.97, BS = 94). Other subclades in the large A-genome clade comprise only minor ribotypes of different accessions of *A. strigosa* (PP = 0.96, BS = 94; PP = 0.95, BS = 94; PP = 0.80, BS = 71), *A. strigosa* + *A. canariensis* (PP = 0.76), and *A. hirtula* (PP = 0.94, BS = 95).

**Figure 3:**
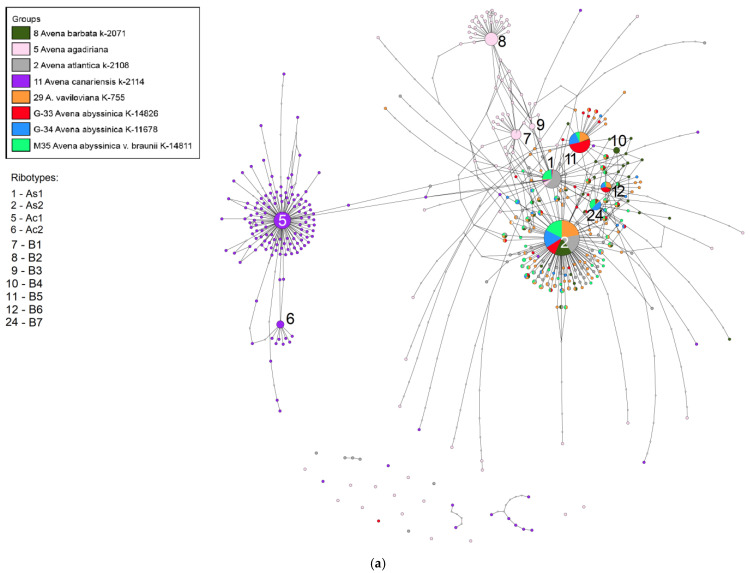

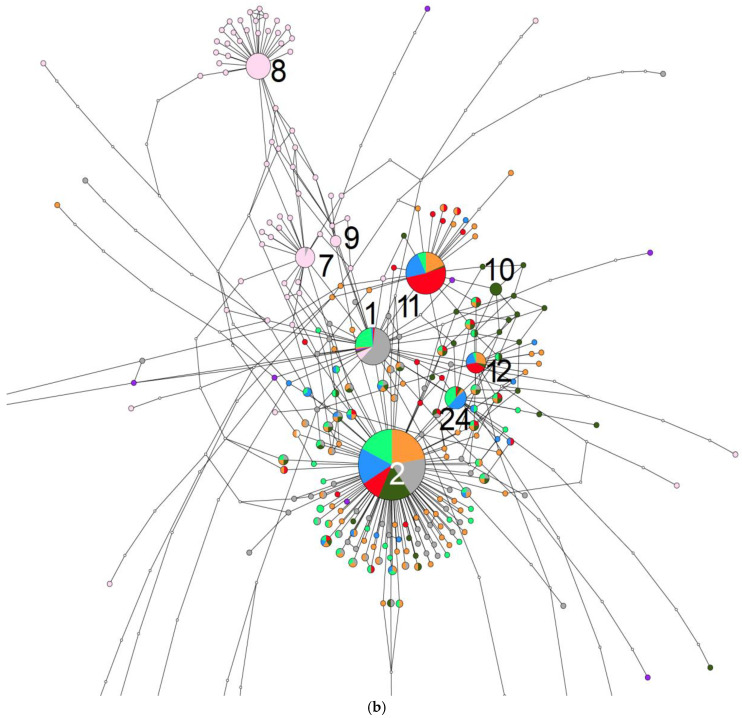
(**a**) Ribotype network of the cultivated *A. abyssinica* and its putative parental taxa. The radius of the circles on the ribotype network is proportional to the percent number of reads for each ribotype, as shown in Table 2. The circles corresponding to the major ribotypes are larger than the others (more than 1000 reads per rDNA pool) and marked with numbers. The smallest circles correspond to ITS1 variants that have been read fewer than 1000 times. (**b**) Ribotype network of the cultivated *A. abyssinica* and its putative parental taxa. A more detailed picture of the major ribotypes.

The main ribotypes of the studied accessions of *A. abyssinica* are different (Figure 3a,b). The main ribotype of *A. abyssinica* var. *braunii* Körn. K-11678 (5337 reads, 35%) and *A. abyssinica* var. *braunii* K-14811 (10866 reads, 49%) are common with the main ribotype of *A. vaviloviana* (Malzev) Mordv. (10884 reads, 47%), the second major ribotype of *A. abyssinica* var. *schimperi* Körn. (3039 reads, 21%), and the main ribotype of *A. atlantica* K-2108 (6353 reads, 39%)—the ribotype As1. The main ribotype of *A. abyssinica* var. *schimperi* Körn. (3291 read, 23%) is identical to the second major ribotype of *A. vaviloviana* (3255 reads, 14%), the second major ribotype of *A. abyssinica* var. *braunii* K-11678 (2387 reads, 16%), and the fourth major ribotype of *A. abyssinica* var. *braunii* K-14811 (1266 reads, 6%). This ribotype we call B5 [22]—it differs from the major ribotypes of the diploids with the A genome and may belong to the B genome. The third major ribotype of *A. abyssinica* var. *schimperi* (1042 reads, 7%) is identical to the third major ribotype of *A. vaviloviana* (1162 reads, 5%), the fourth major ribotype of *A. abyssinica* var. *braunii* K-11678 (1108 reads, 4%), and the fifth major ribotype of *A. abyssinica* var. *braunii* K-14811 (1012 reads, 5%) This ribotype is called B6. The second major ribotype of *A. abyssinica* var. *braunii* K-14811 (4151 read, 19%) is identical to the second major ribotype of *A. atlantica* (4571 read, 28%). This ribotype is called As2. The third major ribotype of *A. abyssinica* var. *braunii* K-11678 (1142 reads, 5%) and the third major ribotype of *A. abyssinica* var. *braunii* K-14811 (2472 reads, 11%) is identical only to the minor ribotype fraction of *A. barbata* and *A. vaviloviana*. This ribotype we call B7.

**Figure 4:**
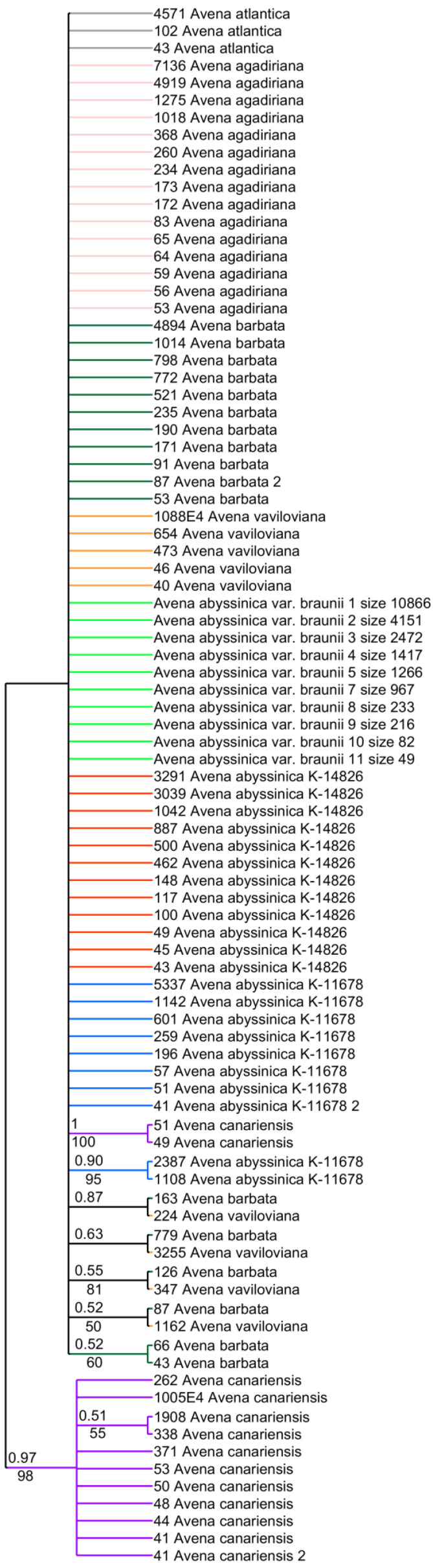
Phylogenetic tree of the ribotypes of the cultivated *A. abyssinica* and its putative parental species built by the Bayesian inference and the maximum likelihood method. The first index above the branch is the posterior probability in the Bayesian inference, and the second index below the branch is the bootstrap index obtained by the maximum likelihood algorithm. When only one index is shown on the branch, it is the posterior probability. The numbers before the species name indicate the number of reads per rDNA pool.

The phylogenetic tree of the ribotypes of *A. abyssinica* and related species shows that all ribotypes of these studied species are A-genome-related (Figure 4). The A- and B-genome-related ribotypes are not strongly distinct from each other, but the *A. canariensis* ribotypes (Ac-genome) are expectedly distinguished into a separate clade (PP = 0.97, BS = 98, Figure 4). The second major ribotype of *A. abyssinica* var. *braunii* K-11678 groups with the third major ribotype of *A. abyssinica* var. *braunii* (PP = 0.9, BS = 95, Figure 4). Additionally, the second major ribotype of *A. vaviloviana* (3255 reads) forms a separate low-supported clade with the minor ribotype of *A. barbata* Pott ex Link (PP = 0.63), the third major ribotype of *A. vaviloviana* groups with one minor ribotype of *A. barbata* (PP = 0.52) as well.

**Figure 5:**
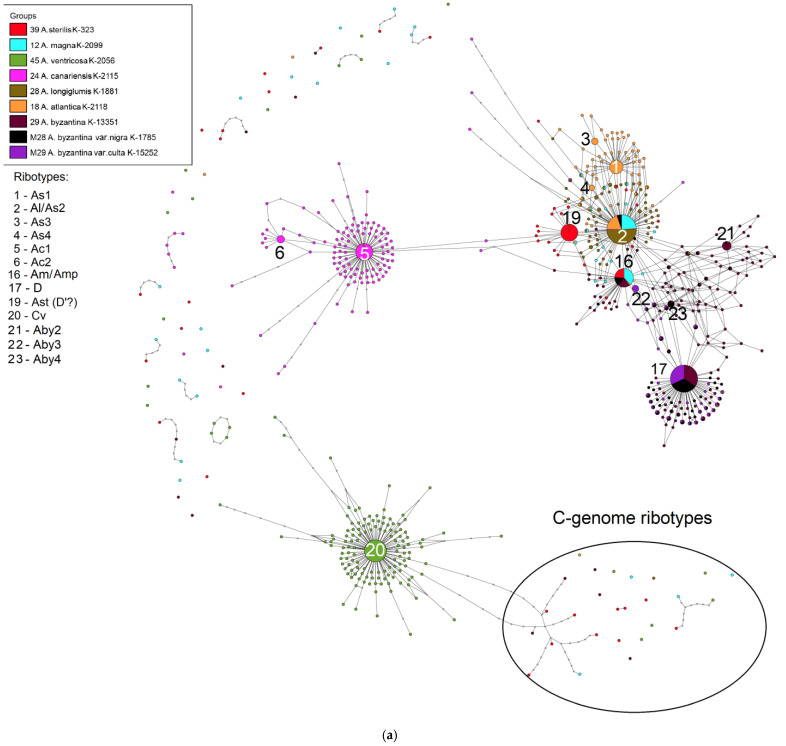

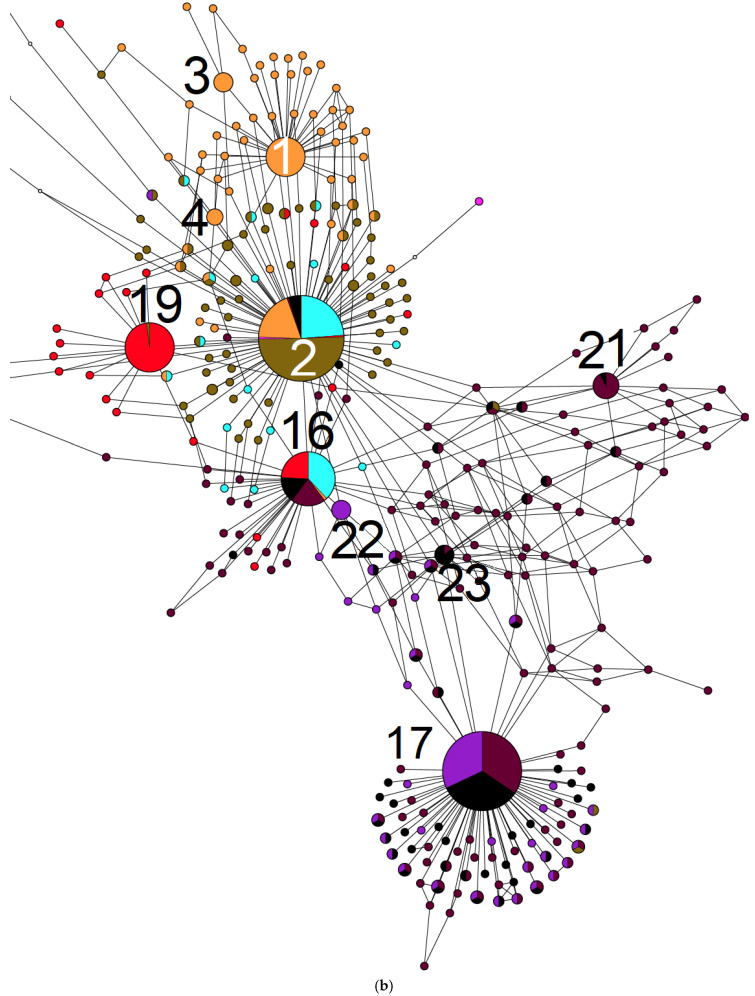
(**a**) Ribotype network of the cultivated *A. byzantina* and its putative parental taxa. The radius of the circles on the ribotype network is proportional to the percent number of reads for each ribotype, as shown in Table 2. The circles corresponding to the major ribotypes are larger than the others (more than 1000 reads per rDNA pool) and marked with numbers. The smallest circles correspond to ITS1 variants that have been read fewer than 1000 times. (**b**) Ribotype network of the cultivated *A. byzantina* and its putative parental taxa. A more detailed picture of the major ribotypes.

The third ribotype network depicts the phylogenetic relationships of the hexaploid *A. byzantina* with the ACD-genome (Figure 5a,b). The main ribotype of all studied accessions of *A. byzantina* (9935 reads, 43%; 7961 read, 42%; 7425 reads, 40%) is specific for the hexaploids. This ribotype belongs to the A-genome family and is not present in any other studied species. We can name it D. The second major ribotype of *A. byzantina* K-13351 (Aby2, 2716 reads, 12%) is shared with the minor ribotype fraction of *A. byzantina* var. *nigra* Mordv. ex Rodionova & Soldatov. The second major ribotype of *A. byzantina* var. *nigra* (1458 reads, 8%) and the third major ribotype of *A. byzantina* K-13351 (2619 reads, 12%) are common with the second major ribotype of *A. sterilis* L. (1820 reads, 14%) and the second major ribotype of *A. magna* K-2099 (3598 reads, 22%), which can be called Am/Amp (major ribotypes of *A. magna* and *A. murphyi* are very close and in one sample they are identical).

The second major ribotype of *A. byzantina* var. *culta* (Thell.) Mordv. (1269 reads, 7%) is specific. This ribotype is Aby3. The third major ribotype of *A. byzantina* var. *nigra* (1054 reads, 6%) is shared with the main ribotype of *A. magna* (5720 reads, 35%), the main ribotype of *A. longiglumis* (9412 reads, 74%), and the second major ribotype of *A. atlantica* K-2118 (6615 reads, 28%). We name this ribotype Al/As2 [22,26]. The fourth major ribotype of *A. byzantina* var. *nigra* (1010 reads, 5%) is common with the minor ribotype of *A. byzantina* K-13351. This ribotype is Aby4. The only minor ribotypes of *A. byzantina* belong to the C-genome family (Figure 5).

**Figure 6:**
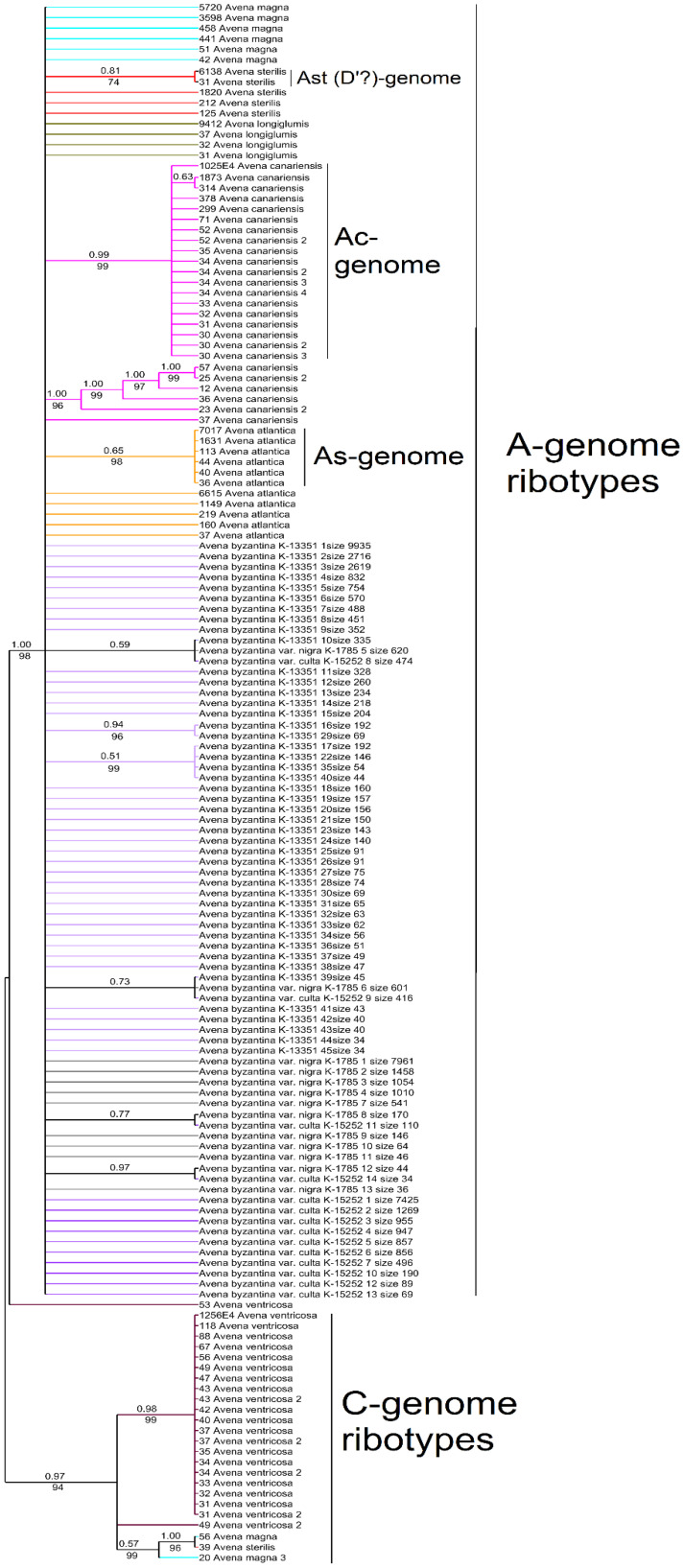
Phylogenetic tree of the ribotypes of the cultivated *A. byzantina* and its putative parental species built by the Bayesian inference and the maximum likelihood method. The first index above the branch is the posterior probability in the Bayesian inference, and the second index below the branch is the bootstrap index obtained by the maximum likelihood algorithm. When only one index is shown on the branch, it is the posterior probability. The numbers before the species name indicate the number of reads per rDNA pool.

On the phylogenetic tree of the ribotypes (threshold is 30 reads per rDNA pool) we see the division of the A- and C-genome ribotype families into separate clades (PP = 0.97, BS = 94 and PP = 1, BS = 98, respectively) (Figure 6). In the A-genome clade we see the subclade that corresponds to the major ribotype of *A. sterilis* with the minor ribotype of *A. sterilis* (PP = 0.81, BS = 74), two subclades of the *A. canariensis* ribotypes (one of them includes major ribotypes of *A. canariensis* (PP = 0.99, BS = 99; PP = 1, BS = 96), and the subclade that comprises the major ribotype of *A. atlantica* (PP = 0.65, BS = 98). The major ribotypes of the accessions of *A. byzantina* do not form subclades in the A-genome clade, while some of the minor ribotypes of *A. byzantina* fall into the separate subclades.

**Figure 7:**
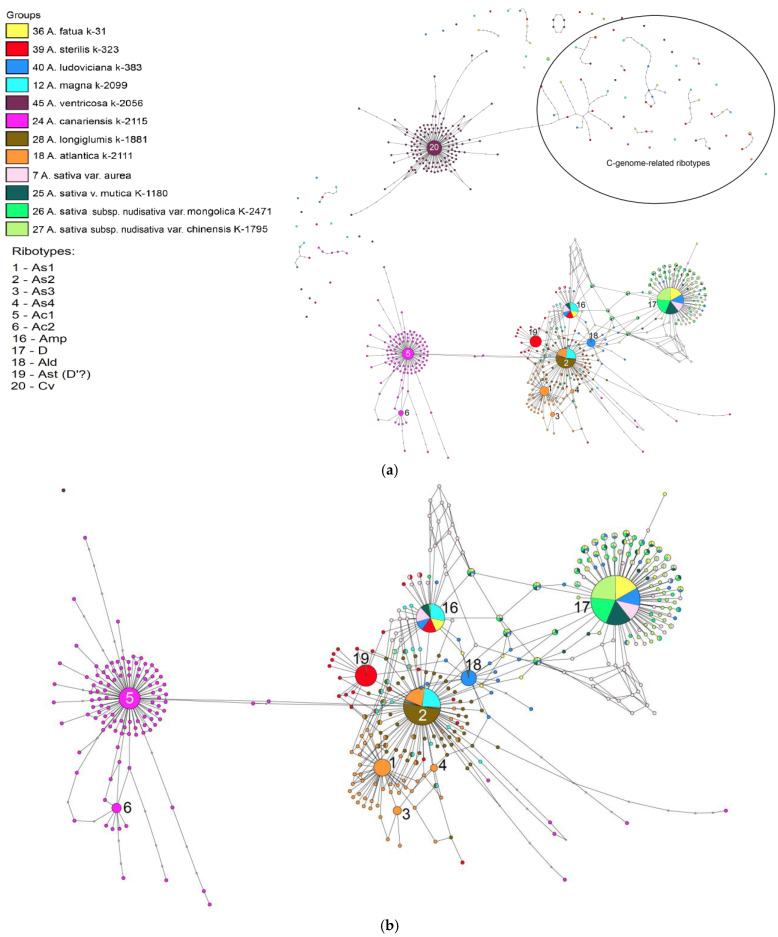
(**a**) Ribotype network of the cultivated *A. sativa* and its putative parental taxa. The radius of the circles on the ribotype network is proportional to the percent number of reads for each ribotype, as shown in Table 2. The circles corresponding to the major ribotypes are larger than the others (more than 1000 reads per rDNA pool) and marked with numbers. The smallest circles correspond to ITS1 variants that have been read fewer than 1000 times. (**b**) Ribotype network of the cultivated *A. sativa* and its putative parental taxa. A more detailed picture of the major ribotypes.

The ribotype network of the hexaploid *A. sativa* and its possible ancestral taxa (Figure 7a,b) is similar to the network constructed by us when we studied the hexaploid wild oats [22]. The main ribotype of all *A. sativa* accessions is common with the main ribotype of *A. fatua* L. (5203 reads, 42%) and *A. ludoviciana* Durieu (4682 reads, 30%). This ribotype was not found in any of the other oats except for a minor fraction of paleopolyploid *A. clauda* Durieu [22]. We call it the D-ribotype [22].

The second major ribotype of *A. sativa* var. *aurea* Körn. (3786 reads, 16%) and of *A. sativa* var. *mutica* Gray (1138 reads, 8%) is identical to the second major ribotype of *A. magna* K-2099 (3598 reads, 22%), *A. fatua* (1608 reads, 13%), and the third major ribotype of *A. ludoviciana* (1409 reads, 9%). This ribotype can be named Am/Amp. Other accessions of *A. sativa—A. sativa* subsp. *nudisativa* var. *mongolica* (Pissarev ex Vavilov) Mordv. and *A. sativa* subsp. *nudisativa* var. *chinensis* (Fisch. ex Roem. et Schult.) Döll—have the second ribotype that is identical to Am/Amp but has fewer than 1000 reads per rDNA pool. As in the wild hexaploids, the C-genome-related ribotypes of the studied accessions of *A. sativa* are present only in a minor quantity and are significantly changed compared with the C-genome ribotypes of *A. ventricosa* Balansa.

**Figure 8:**
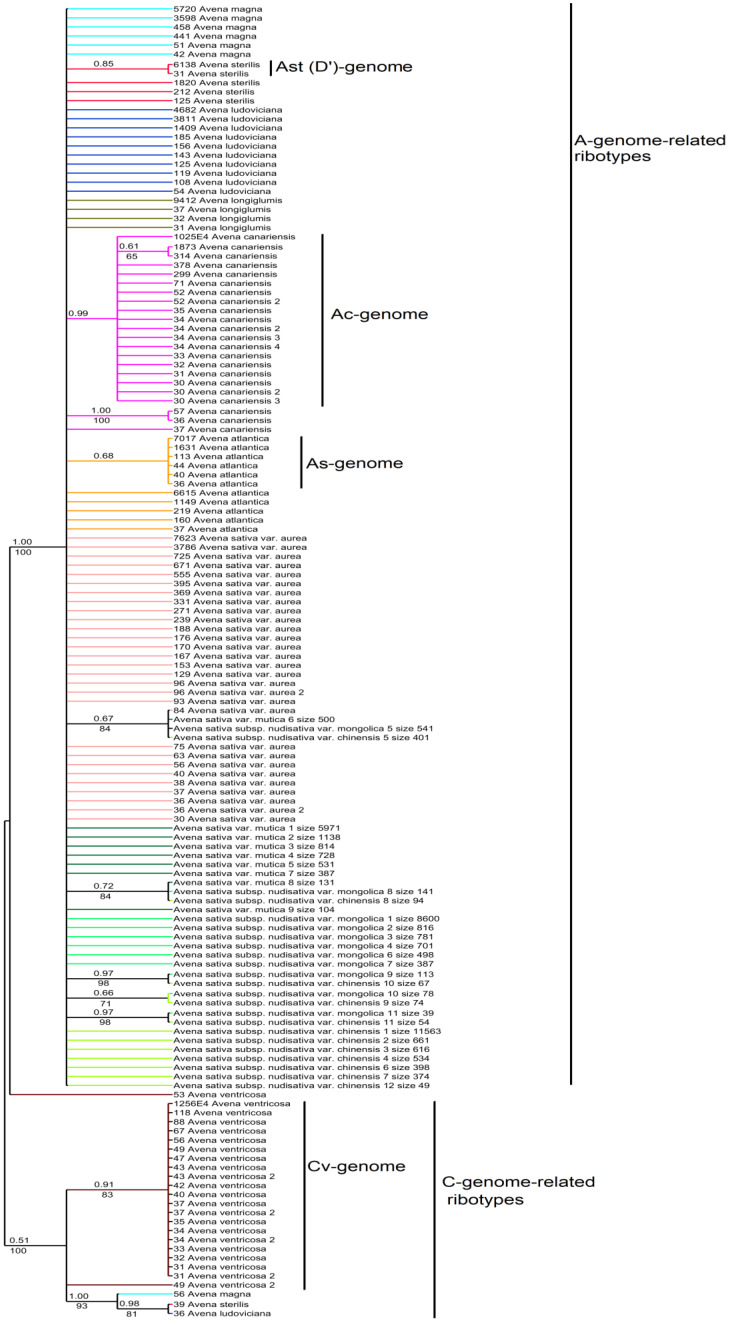
Phylogenetic tree of the ribotypes of the cultivated *A. sativa* and its putative parental species built by the Bayesian inference and the maximum likelihood method. The first index above the branch is the posterior probability in the Bayesian inference, and the second index below the branch is the bootstrap index obtained by the maximum likelihood algorithm. When only one index is shown on the branch, it is the posterior probability. The numbers before the species name indicate the number of reads per rDNA pool.

The ribotype tree (Figure 8) demonstrates distinction of the A- and C-genome-related ribotypes (PP = 1, BS = 100; PP = 0.51, BS = 100, respectively). In the A-genome clade we see the sub-clade that contain Ac-genome (PP = 0.96; PP = 1; BS = 100), the main ribotype of *A. sterilis* (Ast/D’’, PP = 0.85), the major ribotypes of *A. atlantica* (As, PP = 0.68). In addition, minor ribotype fraction of different accessions of *A. sativa* fall into separate subclades.

## 3. Discussion

**Figure 9:**
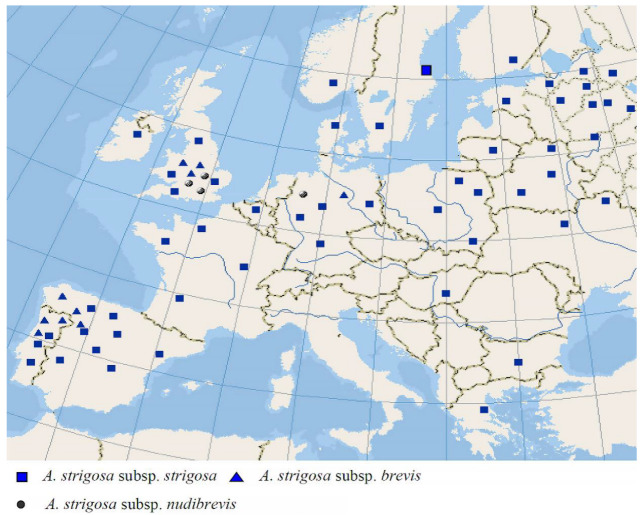
The range of the diploid cultivated species *A. strigosa* (from [2]).

The diploid *A. strigosa* is one of the oldest cultivated species of oats [31]. Unlike many wild and cultivated species, its range is confined to Southern and Middle Europe [2,3] (Figure 9). *Avena strigosa* is widespread throughout most of Europe as a ruderal plant being cultivated before the Second World War [3]. In the Middle Ages, it was cultivated more abundantly [31,32]. This species was described earlier than all the other diploid oats by Johann Schreber [33]. From the karyological point of view, it has the diploid As-karyotype with low heterochromatinization, two metacentric, two submetacentric, one subacrocentric, and two morphologically different satellite (SAT) chromosomes [34]; that is, it is similar to the karyotypes of *A. wiestii* and *A. hirtula*.

However, *A. hirtula* has specific features of its As-karyotype that differ from the chromosome C-banding pattern of *A. strigosa* [34]. Some researchers have regarded *A. strigosa* as a diploid progenitor of the cultivated hexaploid *A. sativa* [35,36,37]. An analysis of the pattern of repetitive sequences has showed the clear difference of *A. strigosa* and *A. sativa* [38]. The sequences of the spacer *psb*A–*trn*H and the gene Acc1 demonstrate the uncertain position of *A. strigosa* among the A-genome-related sequences [18]. A chromosome analysis, on the other hand, suggests that *A. strigosa* can be the A-genomic donor to the hexaploid species [6]. Nevertheless, our NGS data show that, instead, the main ribotype of *A. strigosa* is identical with that of *A. damascena* Rajhathy & B.R.Baum, and is not present in the hexaploids [22,39]. The second and the third major ribotypes in different accessions of *A. strigosa* belong to As2 that is also present in *A. murphyi*—a possible tetraploid progenitor of the hexaploid oats according to the NGS data [22]. These facts may indicate that *A. strigosa* evolved independently from the hexaploid species. Our sequence analysis rather points to a closer relatedness of *A. strigosa* to *A. hirtula* than to the polyploid oats.

**Figure 10:**
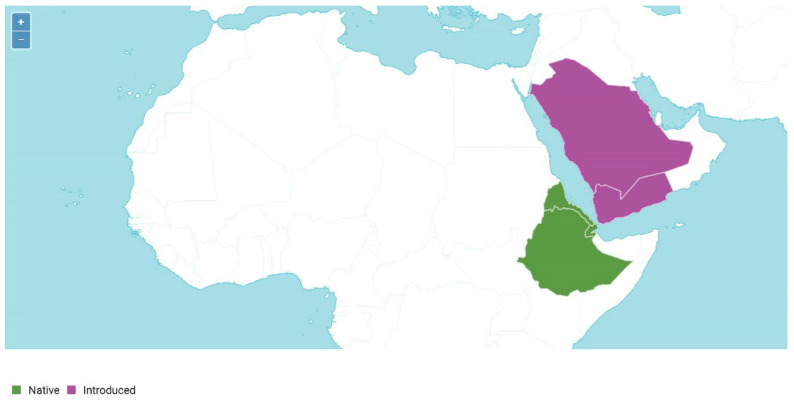
The range of the tetraploid cultivated species *A. abyssinica* (from https://powo.science.kew.org/taxon/urn:lsid:ipni.org:names:391274-1, accessed on 18 March 2025).

*Avena abyssinica* is widespread in Ethiopia, Eritrea, and Yemen ([2], Figure 10). It displaced other grain crops, in particular barley [2]; in the southern parts of the area, *A. abyssinica* is a common segetal weed of wheat. From a morphological point of view, *A. abyssinica* is very close to *A. vaviloviana* and probably replaced it as a cultivated crop [2]. *Avena abyssinica* has two developed awns on the apex of the lemma that is considered to be a rather primitive trait. Two lemma awns are common also for the tetraploid *A. vaviloviana*, *A. barbata*, the diploids *A. clauda*, *A. pilosa* Scop., and the A-genome species except for *A. canariensis*. *Avena abyssinica* has slightly unequal glumes [2] that can be treated as the average value of a trait in evolutionary terms. *Avena abyssinica* has the same karyotype as *A. vaviloviana*, also sharing some of the intergenomic translocations [6]. The tetraploids *A. abyssinica* and *A. vaviloviana* have some significant morphological differences, such as lodicule type, but can freely hybridize in experiments [40]. The NGS data support the hypothesis of a close relationship between *A. abyssinica* and *A. vaviloviana*, most probably *A. abyssinica* is a cultivated derivative of *A. vaviloviana*. The ribotype pattern of these two species is almost the same, though the main ribotypes are different in the studied accessions of *A. abyssinica*. One of the major ribotypes in *A. abyssinica* is As1, that was probably inherited from *A. atlantica*-like ancestors (see [22,39]). The second of the major ribotypes is most probably B-related. One species-specific ribotype of *A. abyssinica* can indicate some evolutionary processes in this cultivated species.

**Figure 11:**
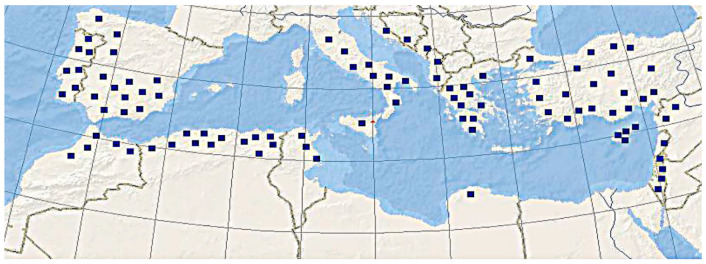
The range of the hexaploid cultivated species *A. byzantina* (from [2]).

*Avena byzantina* belongs to the hexaploid group of oats (2n = 42). Its genome structure is ACD [6,36,41,42,43]. Its natural range covers the Mediterranean (Figure 11). As with the other hexaploid species of *Avena* having in their genomic constitution the C-genome, *A. byzantina* has only a few ribotypes of the minor fraction which may belong to the C-genome (Figure 5a,b). All the major ribotypes of *A. byzantina* are A-related (Figure 5a,b). As with the other hexaploid species, *A. byzantina* has two developed denticles on the lemma which could be a sign of sufficient evolutionary advancement [2]. *Avena byzantina* easily crosses with the other hexaploid oat species [2,44]; its taxonomical placement in many treatments was near *A. sterilis* [45,46,47,48,49,50]. On the other hand, some researchers placed *A. byzantina* as a relative of *A. sativa*, sometimes even as its subspecies [19,51,52]. An analysis of chromosome translocations showed a unique pattern that differed from that of the other hexaploid species [6,43]. Our ribotype sequences indicate that *A. byzantina* could evolve independently from the other hexaploid oats: the studied accessions have species-specific major ribotypes, but its main ribotype is D, as in the other hexaploids. The second major ribotype of *A. byzantina* was probably inherited from the *A. magna/A. murphyi* complex, as in the other studied hexaploids, though one of the *A. byzantina* accessions has the specific second major ribotype. In addition, one of the major ribotypes in the studied accessions of *A. byzantina* is Al/As2, and it is common with *A. atlantica* and *A. magna*. As we see from the NGS data, *A. byzantina* (ACD-genome) is closely related to the other hexaploid species.

**Figure 12:**
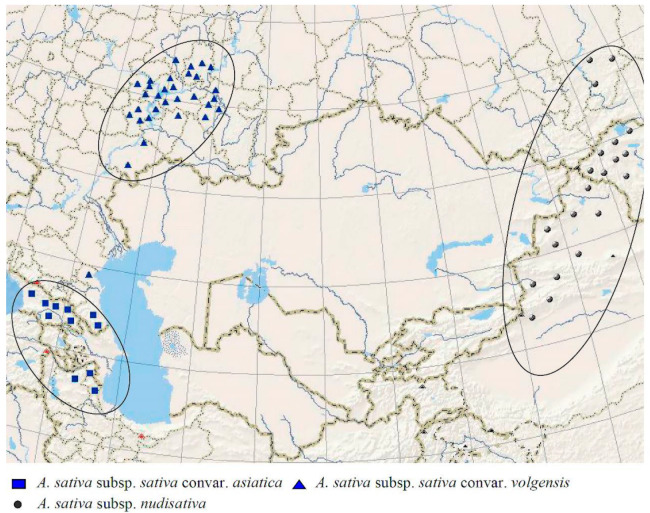
The range of the hexaploid cultivated species *A. sativa* (from [2]).

*Avena sativa* is the most widespread cultivated hexaploid oat (2n = 42). Now, it is cultivated almost all over the world. The possible place of origin of *A. sativa* is Asia, mainly Eastern Asia ([2,48,50,53], Figure 12). From a morphological point of view, *A. sativa* is considered to be the possible derivative of wild *A. fatua*, differing in the degree of the shedding of florets and the pubescence of lemmas [49,52,54,55]. The alternative hypothesis about the origin of *A. sativa* connects it with the wild species of *A. ludoviciana* as its cultivated descendant [2,6]. The karyotype of *A. sativa* is the same as in all other hexaploids, though some researchers have found that three chromosome pairs in the genomes of *A. sativa* and *A. fatua* strongly differ from each other [56]. Our data do not show noticeable difference between *A. sativa* and *A. fatua* in their ribotype structure. The main ribotype of all the studied accessions of *A. sativa* is A-related and probably belongs to the D-subgenome (Figure 7a,b). It is also identical to the main ribotypes of *A. fatua* and *A. ludoviciana* [22]. The second of the major ribotypes in *A. sativa* is also A-related and belongs to the Am/Amp ribotype that is shared with *A. magna* and *A. murphyi* (AC-tetraploids). According to the IGS and RFLP data, *A. insularis* can also be the donor of the A-genome to the *A. sativa*/*A. fatua* complex [57], but we have no rDNA evidences for this. It should be noted that two cultivars of *A. sativa*, *A. sativa* subsp. *nudisativa* var. *mongolica* and *A. sativa* subsp. *nudisativa* var. *chinensis*, do not have the second major ribotype Am/Amp, this ribotype remains in the minor fraction. Additionally, the As1-ribotype that was probably inherited from *A. atlantica* [22] is found in the minor ribotype fraction of *A. sativa* as well as in the other hexaploids. The C-genome-related ribotypes of *A. sativa* (probably from *A. ventricosa*) are found in small quantities (the minor ribotype fraction strongly changed via post-hybridization concerted evolution). Thus, we can assume that *A. sativa* can in fact be the cultivated form of *A. fatua* as was thought previously; *A. sativa* probably went through an additional series of introgression. Both species, *A. sativa* and *A. fatua*, are complex hybrids. The phylogenetic tree constructed on the basis of chloroplast genes has showed that the maternal ancestral taxon for *A. fatua* is *A. murphyi*, and the ancestral taxon for *A. sativa* is *A. sterilis* [58]. Our data, based on NGS, do not contradict these findings, but may show a pattern of relationships along both parental lines. Therefore, *A. murphyi* also appears to be closely related to *A. sativa*, as is *A. fatua*, but it is likely that *A. sativa* and *A. fatua* have different maternal genomes.

**Figure 13:**
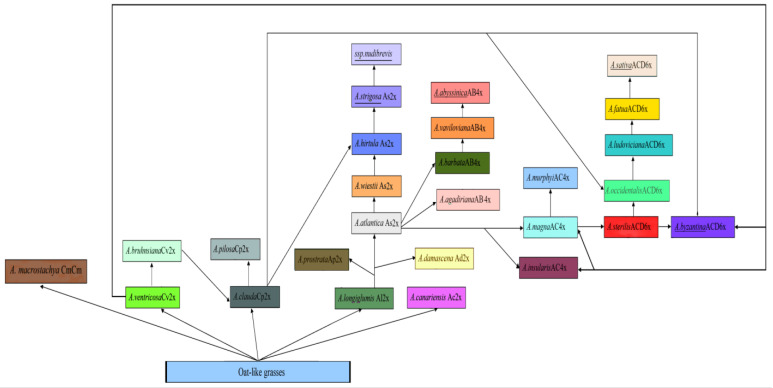
Possible phylogeny of the oat species according to NGS and previous data (morphological and cytogenetic). Cultivated species are underlined.

As a summary of our phylogenetic inferences based on rDNA polymorphism, we can present a brief scheme that reflects the possible evolutionary relationships between all wild and cultivated oat species (Figure 13). This scheme is somewhat more complex than the scheme that was drawn in previous studies [2,3,6]. Based strongly on ITS data, our scheme can, of course, be incomplete and indicate gene evolution rather than genome reorganization. As we see, rDNA being subject to concerted evolution can stay more conservative compared with the genome structure of the species [22]. For example, some modern cytological research of the tetraploid oats set their genome as CD instead of AC [6,18,19]. Our NGS data tell us that the tetraploids with the C-genome could be the AC-genome instead; the D-genome sequences belong to other, different ribotypes. Obviously, this can also be due to chromosome translocations within the ACD-genomes. Our phylogenetic scheme shows the *Avena* taxa with the C-genome as the most primitive, while *A. macrostachya* (autotetraploid) belongs to the separate evolutionary line of the C-genome oats. (Figure 13). *Avena ventricosa* (Cv-genome) (or common ancestor) could give origin to the rare endemic *A. bruhnsiana* Gruner [39,59,60]. Also, *A. ventricosa* could give the Cv-genome to the polyploid *Avena* (AC, ACD) that is very important for understanding the phylogeny of the *Avena* genus (Figure 13). All C-genome remnants of rDNA in the polyploid oats were changed in post-hybridization evolution either by unequal crossing-over or via the “birth-and-death” mechanism [61]. The C-genome diploid donor was most probably a male parent because there were no the C-genome-related chloroplast sequences in the polyploids [18]. However, on top of everything else, an rDNA analysis revealed that the diploid species *A. clauda* having the Cp-genome could be a paleopolyploid (or the ancestral species) which provided the specific A-genome ribotypes to *A. hirtula* (As-genome), and even to the hexaploids *A. fatua* and *A. ludoviciana* (ACD-genome) as the D-genome fraction [22]. The A-genome bearers are probably more evolutionarily developed; they are more represented, and their rDNA dominates in the complex genomes of allopolyploids. Our data supports the previous hypotheses of *A. longiglumis* as the most primitive A-genome oat, and further the origin of *A. hirtula* and *A. strigosa* from *A. longiglumis* [2,3,62,63]. Additionally, we consider *A. prostrata* Ladiz. (Ap) and *A. damascena* (Ad) to be separate lines probably originating from *A. longiglumis* that did not participate in the formation of the cultivated polyploids. The diploid *A. canariensis* (Ac) is rather distant from the other oat species; nevertheless, it could hybridize with the As-genome species in some earlier time; its ribotype fraction of the As-genome was found in very small quantities. For example, some minor ribotypes of *A. canariensis* group with the minor ribotypes of *A. strigosa* (Figure 2a,b), and the minor ribotype of *A. canariensis* is identical with the major ribotype of *A. atlantica*, Al/As2 (Figure 7a,b). We have not found any specific rDNA of *A. canariensis* in the tetraploid *A. agadiriana*, as was supposed by some authors [6], though one minor ribotype of *A. canariensis* is identical with the major ribotype Al/As2. We see that *A. agadiriana* has an unusual genomic constitution compared with the other polyploidy oats, though we cannot clearly identify the autotetraploid status of the studied accessions of *A. agadiriana* [22]. One of its major ribotypes is Al/As2, as is the case for allotetraploids with the C-genome [22]. We treat the cultivated *A. abyssinica* (AB) as a probable descendant of *A. vaviloviana*, as was previously found by morphological criteria [49,64]. According to our NGS data, *A. magna* and *A. murphyi* are very closely related, and their major ribotypes probably were later inherited by the hexaploid species. Some accessions of *A. magna* have their own specific ribotypes. We confirm the origin of the C-genome in the AC-tetraploids from *A. ventricosa*, which was assumed previously [7]. The hexaploid species have the most complicated evolutionary history [2,3,6]. According to the morphological data, the first hexaploid species in the genus *Avena* was *A. sterilis* [2,64,65]. Then, after a series of mutations of the dispersal of caryopsis, a cultivated species *A. byzantina* and a wild *A. occidentalis* Durieu appeared [2]. Our NGS data has confirmed this hypothesis [22]. In addition, surprisingly, we see a possible participation of *A. clauda* in the formation of the D-genome of the hexaploids [22]. It is an unusual fact because, from the karyological point of view, *A. clauda* is a typical diploid with the Cp-genome type [7]. The probable D-genome ribotypes were A-related that supported the previous assumptions about the D-genome origin. Chloroplast sequence studies supposed that *A. murphyi* and *A. maroccana* (=*A. magna*) provided the D-subgenome to the polyploids [18]. However, our sequence data shows two major A-related ribotypes in the hexaploids *A. occidentalis*, *A. ludoviciana*, *A. fatua*, and *A. sativa*. One of the A-related ribotypes was identical to that of *A. murphyi* and *A. magna*, whereas the other was specific to this group of the hexaploids (Figure 7a,b) and *A. clauda* [22]. The AC-genome-related ITS1 sequences could persist in the CD-genome oats because of the translocation events detected by Yan et al. [12] when the A-genome chromosomes were transformed into the D-genome chromosomes after a series of introgressive hybridizations. Previous studies have not found any living D-genome diploid, but we can assume that *A. clauda* was either a paleohomoploid hybrid with the D-genome from extinct species or there was a specific D-genome mutation in its rDNA. Our NGS analysis supports the earlier hypotheses on the origin of *A. sativa* from *A. fatua* [49,52,55]. *Avena sativa* could originate from *A. fatua* via specific “sativa-mutation” [2] being originally a weed in barley and wheat crops.

## 4. Materials and Methods

### 4.1. Plant Materials

We analyzed four cultivated species of *Avena*, *A. strigosa, A. abyssinica, A. byzantina*, and *A. sativa*. Material was taken from the Federal Research Center N. I. Vavilov All-Russian Institute of Plant Genetic Resources (VIR). Plant samples were determined by the curator of the world oat collection of VIR, I. G. Loskutov and senior researcher A. A. Gnutikov. In this paper, the studied accessions are considered not only at the species level, but at the subspecies level and for botanical varieties [1,2,3]. To construct phylogenetic hypotheses, we took the putative wild parental species and relatives of the cultivated oats. The list of the studied cultivated species is presented in Table 1.

### 4.2. DNA Extraction, Amplification, and Sequencing

Genomic DNA was extracted from dried leaf and seed material using a Qiagen Plant Mini Kit (Qiagen Inc., Hilden, Germany).

For amplification of the 18S–ITS1–5.8S rDNA region, the following conditions were used: initial denaturation at 94 °C for 1 min, followed by 25 cycles of 94 °C for 30 s, 55 °C for 30 s, 72 °C for 30 s, and a final elongation of 72 °C for 5 min using ITS 1P [66] and ITS 2 [67] primers. PCR was carried out in 15 μL of the reaction mixture containing 0.5–1 unit of activity of Q5^®^ High-Fidelity DNA Polymerase (NEB, Ipswich, MA, USA), 5 pM of forward and reverse primers, 10 ng of DNA template, and 2 nM of each dNTP (Life Technologies, Thermo Fisher Scientific, Waltham, MA, USA). The PCR products were then purified according to the Illumina recommended method using AMPureXP kit (Beckman Coulter, Indianapolis, IN, USA).

The libraries for sequencing were prepared according to the manufacturer’s MiSeq Reagent Kit Preparation Guide (Illumina) (https://www.illumina.com/products/by-type/sequencing-kits/cluster-gen-sequencing-reagents/miseq-reagent-kit-v3.html (accessed on 11 May 2020)). They were sequenced on an Illumina MiSeq instrument (Illumina, San Diego, CA, USA) using a MiSeq^®^ Reagent Kit v3 (600 cycles) with paired-end reading (2 × 300 *n*) following the manufacturer’s instructions. The fragments were amplified and sequenced at the Center for Shared Use “Genomic Technologies, Proteomics, and Cell Biology” of the All-Russian Research Institute of Agricultural Microbiology.

The sequences were trimmed with the Trimmomatic [68], included in Unipro UGENE 41.0 [69] using the following parameters: PE reads, sliding window trimming with size 4, quality threshold 12, and minimal read length 130. Further, paired sequences were combined, dereplicated, and sorted into the ribotypes with the aid of vsearch 2.7.1 [70]. Sequence alignment was performed by the MUSCLE algorithm [71] included in MEGA XI [72]. The ribotypes are the sequences with a certain amount of reads per rDNA pool.

### 4.3. Data Analysis

We constructed a hybrid network of the ribotypes with a certain number of reads within the rDNA pool of the species using TCS 1.21 [73], and visualized it in TCSBU 1.1 [74]. The number of sequences with read counts greater than 1000 was increased by the percentage of reads of these sequences relative to the total number of reads. This was performed to visualize the composition of ribotypes, so that the contribution of each sample to the major ribotype is reflected. The resulting network shows the possible origin of the species via multiple hybridization according to the statistical parsimony algorithm. We set a threshold for this analysis to 10 reads per rDNA pool. In addition, we inferred the ribotype trees by the Bayesian and the maximum likelihood methods. The threshold for tree inference was 30 reads per the rDNA pool. An evolutionary model for the ribotype sequences was computed by jModelTest 2.1.7 [75]. The GTR+I+G model was selected as the best fit according to the Akaike information criterion (AIC) values. The Bayesian inference was performed by MrBayes 3.2.2 [76] as follows: 7–13 millions of generations, sampling trees every 100 generations, the first 25% of trees were discarded as burn-in. The ML search was performed by IQ-TREE 1.6.12 [77] under the ultrafast bootstrap option of 1000 generations. In this case, the threshold was 30 reads per rDNA pool.

## 5. Conclusions

As we see, many assumptions on the origin of the cultivated oats made earlier on the base of morphological and cytogenetic data are confirmed and clarified by our molecular data received by NGS. At the same time, they evidently revealed some hidden cases of hybridization. The A- and C-subgenome of the oat species, even with strong morphological and karyological differences, could in the past intercross and pass the further stages of introgression producing new stable combinations of genomes. Most species of *Avena* could (at least partly) form an introgressive interspecies complex as a more or less complete evolutionary unit. The discovery of the involvement of the paleohomoploid hybrid *A. clauda* in the evolution of some hexaploid oats and the isolation of *A. prostrata* and *A. damascena* into evolutionary lineages that did not participate in the formation of cultivated polyploids tetraploids are quite non-trivial, previously undescribed phenomena. Undoubtedly, new approaches and data at the genomic and/or metagenomic level will be able to shed more light on the phylogenetic picture in the complex, hybridizable plant genus *Avena*.

## Figures and Tables

**Table 1:** Summary of the cultivated oat species used in the present study.

Species	Sample ID	Country of Origin	Accession Number
*Avena strigosa* subsp. *brevis* var. *candida*	K-4480	United Kingdom: Wales	PV391964–PV392013
*Avena strigosa* var. *strigosa*	K-15025	Canada	PV392014–PV392035
*Avena strigosa* subsp. *nudibrevis*	K-14943	United Kingdom	PV392036–PV392071
*Avena strigosa* subsp. *brevis* var. *candida*	K-5233	Portugal: Aveiro, north from Coimbra	PV392072–PV392136
*Avena strigosa* var. *strigosa*	K-5195	Spain: Los Nogales, Lugo, Galicia	PV392137–PV392197
*Avena abyssinica* var. *schimperi*	K-14826	Ethiopia	PV392232–PV392262
*Avena abyssinica* var. *braunii*	K-11678	Ethiopia	PV392263–PV392291
*Avena abyssinica* var. *braunii*	K-14811	Ethiopia	PV392292–PV392371
*Avena byzantina*	K-13351	Spain	PV397121–PV397264
*Avena byzantina* var. *nigra*	K-1785	USA	PV397265–PV397313
*Avena byzantina* var. *culta*	K-15252	Tunisia	PV397314–PV397353
*Avena sativa* var. *aurea*	K-14787	Russia: Moscow Oblast	PV429768–PV429858
*Avena sativa* var. *mutica*	K-1180	Russia: Tambov Oblast	PV429859–PV429881
*Avena sativa* subsp. *nudisativa* var. *mongolica*	K-2471	Mongolia	PV429882–PV429916
*Avena sativa* subsp. *nudisativa* var. *chinensis*	K-1795	USA	PV429917–PV429978

**Table 2:** Summary of the major ribotypes of cultivated oats (more than 1000 reads per rDNA pool).

Species	Genome	Total Number of Reads	Ribotype Symbol (of the Major Ribotypes)	Number of Reads	% from the Total Number of Reads
*Avena strigosa* subsp. *brevis* var. *candida*	As	21,927	Ad2/As3	8781	40
			Al/As2	2412	11
			As5	2325	11
			As6	1703	8
*Avena strigosa* var. *strigosa*	As	15,373	Ad2/As3	6572	43
			Al/As2	1691	11
			As5	1543	10
			As6	1220	8
*Avena strigosa* subsp. *nudibrevis*	As	20,746	Ad2/As3	9087	44
			Al/As2	2282	11
			As5	2135	10
			As6	1628	8
*Avena strigosa* subsp. *brevis* var. *candida*	As	24,714	Ad2/As3	7977	32
			As5	3707	15
			Al/As2	2962	12
			As6	1995	8
*Avena strigosa* var. *strigosa*	As	15,576	Ad2/As3	9069	58
			As5	4517	29
			Al/As2	3642	23
			As6	2493	16
*Avena abyssinica* var. *schimperi*	AB	14,399	B5	3291	23
			As1	3039	21
			B6	1042	7
*Avena abyssinica* var. *braunii* K-11678	AB	15,270	As1	5337	35
			B5	2387	16
			B7	1142	5
			B6	1108	4
*Avena abyssinica* var. *braunii* K-14811	AB	21,862	As1	10,877	49
			As2	4151	19
			B7	2472	11
			B5	1266	6
			B6	1012	5
*Avena byzantina*	ACD	23,292	D	9935	43
			Aby2	2716	12
			Am/Amp	2619	12
*Avena byzantina* var. *nigra*	ACD	18,956	D	7961	42
			Am/Amp	1458	8
			Al/As2	1054	6
			Aby4	1010	5
*Avena byzantina* var. *culta*	ACD	18,761	D	7425	40
			Aby3	1269	7
*Avena sativa* var. *aurea*	ACD	23,663	D	7623	32
			Am/Amp	3786	16
*Avena sativa* var. *mutica*	ACD	13,886	D	5971	43
			Am/Amp	1138	8
*Avena sativa* subsp. *nudisativa* var. *mongolica*	ACD	16,539	D	8600	52
*Avena sativa* subsp. *nudisativa* var. *chinensis*	ACD	19,272	D	11,563	60

**Table 3:** Primary structure of the major ribotypes (more than 1000 reads per rDNA pool) obtained by NGS. The numbers show the position in the alignment of the major ribotypes. D is a deletion.

									1	1	1	1	1	1	1	1	1	1	1	1	1	1	1	1	1	1	1	1	2	2	2	2	2	2	2	2	2	2	2	2	2	2	2	2	2	2	2	3	3	3
	2	3	4	5	6	8	9	9	0	1	1	1	1	2	2	3	3	3	3	4	6	7	7	7	7	8	9	9	0	0	0	1	2	2	3	4	4	4	4	5	5	5	6	6	6	7	7	0	0	1
	9	6	3	6	2	1	4	6	2	1	5	6	8	2	6	0	5	7	8	3	1	1	4	5	7	4	4	8	3	4	8	4	3	5	7	0	3	4	7	2	7	9	2	5	9	7	9	4	7	6
As1	C	A	C	G	G	G	C	A	C	T	T	G	T	T	G	A	D	T	C	A	C	T	A	G	G	T	G	C	G	C	G	G	C	C	T	G	C	A	A	C	T	G	G	C	T	A	G	C	D	T
Al/As2	.	.	.	.	.	.	.	.	.	.	.	.	.	.	.	C	D	.	.	.	.	.	.	.	.	.	.	.	.	.	.	.	.	.	.	.	.	.	.	.	.	.	.	.	.	.	.	.	D	.
Ad2/As3	.	.	.	.	.	.	.	.	.	.	.	.	.	.	.	.	D	.	T	.	.	.	.	.	.	.	.	.	.	.	.	.	.	.	.	.	.	.	.	.	.	.	.	.	.	.	.	.	D	.
As5	.	.	.	.	.	.	.	.	.	.	.	.	.	.	.	C	D	.	.	.	.	.	.	T	.	.	.	.	.	.	.	.	.	.	.	.	.	.	.	.	.	.	.	.	.	.	.	.	D	.
As6	.	.	.	.	.	.	.	.	.	.	.	.	.	.	.	.	D	.	T	.	.	.	.	T	.	.	.	.	.	.	.	.	.	.	.	.	.	.	.	.	.	.	.	.	.	.	.	.	D	.
B5	.	.	.	.	.	.	.	.	.	.	.	.	.	.	.	C	D	.	.	.	.	.	.	.	.	.	.	.	A	.	.	.	.	.	.	.	.	.	.	.	.	.	.	.	.	.	.	.	D	.
B6	.	.	.	.	.	.	.	.	.	.	.	.	.	.	.	.	D	.	.	.	.	.	.	T	.	.	.	.	.	.	.	.	.	.	.	.	.	.	.	.	.	.	.	.	.	.	.	.	D	.
B7	.	.	.	.	.	.	.	.	.	.	.	.	.	.	.	T	D	.	.	.	.	.	.	.	.	.	.	.	.	.	.	.	.	.	.	.	.	.	.	.	.	.	.	.	.	.	.	.	D	.
D	.	.	.	.	.	.	.	.	.	.	.	A	.	.	.	C	D	.	.	.	.	C	.	.	.	.	.	.	.	.	.	.	.	.	.	.	.	.	.	.	.	.	.	.	.	.	.	.	D	.
Aby2	.	.	.	.	.	.	.	.	.	.	A	.	.	.	.	C	D	.	T	.	.	.	.	.	.	.	.	.	.	.	.	.	.	.	.	.	.	.	.	.	.	.	.	.	.	.	.	.	D	.
Aby3	.	.	.	.	.	.	.	.	.	.	.	.	.	.	.	C	D	C	.	.	.	.	.	.	.	.	.	.	.	.	.	.	.	.	.	.	.	.	.	.	.	.	.	.	.	.	.	.	D	.
Aby4	.	.	.	.	.	.	.	.	.	.	.	.	.	.	.	C	D	.	.	.	.	C	.	.	.	.	.	.	.	.	.	.	.	.	.	.	.	.	.	.	.	.	.	.	.	.	.	.	D	.
Am/Amp	.	.	.	.	.	.	.	.	.	.	.	.	.	.	.	C	D	.	.	.	.	.	.	.	T	.	.	.	.	.	.	.	.	.	.	.	.	.	.	.	.	.	.	.	.	.	.	.	D	.
A. byzantina C-genome-related	T	G	T	A	T	A	T	T	T	D	D	.	C	C	A	C	C	.	T	G	A	C	G	A	.	C	T	A	A	T	A	A	T	T	C	T	T	G	G	A	C	T	A	T	G	C	A	D	C	C

## Data Availability

The original contributions presented in this study are included in the article. Further inquiries can be directed to the corresponding author.
